# Developing a National-Scale Exposure Index for Combined Environmental Hazards and Social Stressors and Applications to the Environmental Influences on Child Health Outcomes (ECHO) Cohort

**DOI:** 10.3390/ijerph20146339

**Published:** 2023-07-10

**Authors:** Sheena E. Martenies, Mingyu Zhang, Anne E. Corrigan, Anton Kvit, Timothy Shields, William Wheaton, Deana Around Him, Judy Aschner, Maria M. Talavera-Barber, Emily S. Barrett, Theresa M. Bastain, Casper Bendixsen, Carrie V. Breton, Nicole R. Bush, Ferdinand Cacho, Carlos A. Camargo, Kecia N. Carroll, Brian S. Carter, Andrea E. Cassidy-Bushrow, Whitney Cowell, Lisa A. Croen, Dana Dabelea, Cristiane S. Duarte, Anne L. Dunlop, Todd M. Everson, Rima Habre, Tina V. Hartert, Jennifer B. Helderman, Alison E. Hipwell, Margaret R. Karagas, Barry M. Lester, Kaja Z. LeWinn, Sheryl Magzamen, Rachel Morello-Frosch, Thomas G. O’Connor, Amy M. Padula, Michael Petriello, Sheela Sathyanarayana, Joseph B. Stanford, Tracey J. Woodruff, Rosalind J. Wright, Amii M. Kress

**Affiliations:** 1 Department of Kinesiology and Community Health, University of Illinois Urbana-Champaign, Urbana, IL 61801, USA; 2 Department of Epidemiology, Johns Hopkins University Bloomberg School of Public Health, Baltimore, MD 21205, USA; 3 Research Triangle Institute, Research Triangle Park, NC 27709, USA; 4 Child Trends, Bethesda, MD 20814, USA; 5 Department of Pediatrics, Hackensack Meridian School of Medicine, Nutley, NJ 07110, USA; 6 Department of Pediatrics, Albert Einstein College of Medicine, Bronx, NY 10461, USA; 7 Avera Research Institute, Sioux Falls, SD 57105, USA; 8 Department of Biostatistics and Epidemiology, Rutgers School of Public Health, Piscataway, NJ 08854, USA; 9 Department of Population and Public Health Sciences, Keck School of Medicine, University of Southern California, Los Angeles, CA 90033, USA; 10 Marshfield Clinic Research Institute, Marshfield, WI 54449, USA; 11 Department of Psychiatry and Behavioral Sciences, University of California San Francisco, San Francisco, CA 94143, USA; 12 Department of Pediatrics, University of California San Francisco, San Francisco, CA 94143, USA; 13 Department of Pediatrics, Vanderbilt University Medical Center, Nashville, TN 37232, USA; 14 Massachusetts General Hospital, Harvard Medical School, Boston, MA 02114, USA; 15 Department of Pediatrics, The Icahn School of Medicine at Mount Sinai, New York, NY 10029, USA; 16 Department of Pediatrics-Neonatology, Children’s Mercy Hospital, Kansas City, MO 64108, USA; 17 Department of Public Health Sciences, Henry Ford Health, Detroit, MI 48202, USA; 18 Department of Pediatrics, New York University Grossman School of Medicine, New York, NY 10016, USA; 19 Division of Research, Kaiser Permanente Northern California, Oakland, CA 94612, USA; lisa.a.croen@kp.org; 20 Lifecourse Epidemiology of Adiposity and Diabetes (LEAD) Center, University of Colorado Anschutz Medical Campus, Aurora, CO 80045, USA; 21 New York State Psychiatric Institute, Columbia University, New York, NY 10032, USA; 22 Department of Obstetrics and Gynecology, Emory University School of Medicine, Atlanta, GA 30322, USA; 23 Gangarosa Department of Environmental Health, Emory University Rollins School of Public Health, Atlanta, GA 30322, USA; 24 Department of Medicine, Vanderbilt University School of Medicine, Nashville, TN 37203, USA; 25 Department of Pediatrics, Wake Forest School of Medicine, Winston-Salem, NC 27101, USA; 26 Department of Psychiatry, University of Pittsburgh, Pittsburgh, PA 15213, USA; 27 Department of Epidemiology, Geisel School of Medicine, Dartmouth College, Lebanon, NH 03756, USA; 28 Department of Psychiatry and Human Behavior, Alpert Medical School of Brown University, Providence, RI 02903, USA; 29 Department of Environmental and Radiological Health Sciences, Colorado State University, Fort Collins, CO 80523, USA; 30 Department of Environmental Science, Policy and Management and School of Public Health, University of California Berkeley, Berkeley, CA 94720, USA; 31 Departments of Psychiatry, Psychology, Neuroscience, and Obstetrics and Gynecology, University of Rochester Medical Center, Rochester, NY 41642, USA; 32 Department of Obstetrics, Gynecology and Reproductive Sciences, University of California San Francisco, San Francisco, CA 94158, USA; 33 Institute of Environmental Health Sciences and Department of Pharmacology, Wayne State University, Detroit, MI 48202, USA; 34 Seattle Children’s Research Institute, Seattle, WA 98105, USA; 35 Department of Pediatrics, University of Washington, Seattle, WA 98195, USA; 36 Department of Pediatrics, Family and Preventive Medicine, University of Utah School of Medicine, Salt Lake City, UT 84132, USA

**Keywords:** neighborhoods, environmental hazards, social stressors, health disparities

## Abstract

Tools for assessing multiple exposures across several domains (e.g., physical, chemical, and social) are of growing importance in social and environmental epidemiology because of their value in uncovering disparities and their impact on health outcomes. Here we describe work done within the Environmental influences on Child Health Outcomes (ECHO)-wide Cohort Study to build a combined exposure index. Our index considered both environmental hazards and social stressors simultaneously with national coverage for a 10-year period. Our goal was to build this index and demonstrate its utility for assessing differences in exposure for pregnancies enrolled in the ECHO-wide Cohort Study. Our unitless combined exposure index, which collapses census-tract level data into a single relative measure of exposure ranging from 0–1 (where higher values indicate higher exposure to hazards), includes indicators for major air pollutants and air toxics, features of the built environment, traffic exposures, and social determinants of health (e.g., lower educational attainment) drawn from existing data sources. We observed temporal and geographic variations in index values, with exposures being highest among participants living in the West and Northeast regions. Pregnant people who identified as Black or Hispanic (of any race) were at higher risk of living in a “high” exposure census tract (defined as an index value above 0.5) relative to those who identified as White or non-Hispanic. Index values were also higher for pregnant people with lower educational attainment. Several recommendations follow from our work, including that environmental and social stressor datasets with higher spatial and temporal resolutions are needed to ensure index-based tools fully capture the total environmental context.

## 1. Introduction

The use of tools and methods to assess multiple exposures that jointly impact health outcomes is an area of rapid growth in the field of environmental health. The National Institute of Environmental Health Sciences has identified the examination of the effects of co-exposures on health as a current priority [1], noting that environmental exposures do not exist in isolation and have the potential to interact in unexpected ways. Several tools have been developed to characterize how multiple environmental or social stressors may contribute to disparities in health. Examples include EJSCREEN [2], CalEnviroScreen [3], the Social Vulnerability Index (SVI) [4], and the Child Opportunity Index [5]. Most recently, the Agency for Toxic Substances and Disease Registry (ATSDR) released the Environmental Justice Index (EJI) 2022, which includes indicators of social stressors and environmental hazards along with community-level health indicators [6]. In 2022, The Council on Environmental Quality released its Climate and Economic Justice Screening Tool to assess cumulative impacts from environmental and social stressors and to designate disadvantaged communities for the targeting of investments from President Biden’s Justice40 Initiative [7]. Other tools have been developed to provide area-specific information for states and metropolitan areas [8,9,10,11,12].

There are several challenges to developing indices that account for the multitude of exposures experienced at the neighborhood or residential level. Depending on data availability, some tools can include several environmental and social indicators of neighborhood quality. For example, CalEnviroScreen 3.0 and 4.0 include data on particulate matter and ozone concentrations, pesticide use, drinking water contamination, childhood lead exposures, and a number of neighborhood-level socioeconomic indicators, among others [3,13]. However, these data are limited to California census tracts only. Key inputs, such as the pesticide use registry, are unavailable elsewhere. Other tools have excellent spatial coverage but tend to focus on only one domain. For example, the SVI covers most census tracts in the United States (U.S.) and includes indicators of neighborhood socioeconomic status (SES) such as poverty and crowded housing, but does not consider environmental exposures [4]. Both the EJI and the Climate and Economic Justice Screening Tool include a variety of social, environmental, and health indicators, but data for many indicators are not available prior to 2020 [6]. Similarly, the Environmental Protection Agency (EPA) EJSCREEN tool has integrated national-level data on environmental hazards and social stressors to examine issues of disproportionate exposures and environmental injustice in the U.S., but the tool does not provide a composite measure of cumulative exposure; while users may view data on one or more environmental hazards, the tool is limited in its temporal coverage [2]. There are limited existing options that will facilitate investigations of stressors in both the environmental and social domains using a single integrated framework across multiple years and the entire contiguous U.S. Such studies would require comprehensive data with complete geographic and temporal coverage for spatial units that are resolved enough to highlight gradients in exposure within populations.

Nevertheless, such tools can be helpful for examining inequalities in exposure and prioritizing resource deployment. Additionally, indices that characterize multiple stressors have been used as combined exposure variables in epidemiological studies. Because these index-based methods capture multiple exposures at a time, they are useful for exploring the “total” environmental context and how it contributes to health outcomes. For example, higher CalEnviroScreen cumulative impact scores have been linked to poorer ovarian cancer survival [14], higher asthma-related hospitalization rates among children [15], and reduced lung function among patients with idiopathic pulmonary fibrosis [16]. Likewise, higher SVI scores have been associated with higher heat-related emergency department visits [17] and a higher risk of postoperative complications among cancer patients [18]. Importantly, indices of environmental or social stressors have been used to explore associations between prenatal exposure and adverse outcomes such as congenital heart disease [19] and sudden unexpected infant death [20] and measures such as gestational age and birth weight [21]. However, investigations of associations between index-based exposures during the prenatal period and other perinatal or childhood health outcomes are limited.

There is growing interest in understanding how multiple environmental hazards and social stressors experienced during the sensitive prenatal and early life periods impact children’s health and well-being. The Environmental influences on Child Health Outcomes (ECHO) Program, which is sponsored by the National Institutes of Health (NIH) Office of the Director, provides an excellent opportunity to expand this body of work by leveraging the ECHO-wide Cohort Study, a collaboration of 69 cohorts that includes pregnant people and children enrolled in studies across the U.S. [22]. Because there are limited tools for assessing both environmental hazards and social stressors in a single framework with adequate spatial and temporal coverage for the ECHO-wide cohort, we sought to develop a national-level index that would incorporate data across multiple environmental and social domains with appropriate temporal coverage and application in multiple studies. Our goal was to capture indicators of environmental and social factors at the neighborhood level that would have relevance to the prenatal and early-life periods. We defined environmental hazards as features of the chemical or built environment that could potentially harm health (e.g., air pollution and lack of green space). We defined social stressors as neighborhood-level social determinants of health that reflect constructs such as lower socioeconomic status and social vulnerability. Additionally, a national data set with spatial and temporal coverage would facilitate regional analyses wherein we can explore how environmental and social determinants interact in geographically and culturally different regions of the country.

Our objective was to create a single exposure index that combined available data on several environmental and social indicators at the national level to facilitate epidemiology studies for the ECHO-wide Cohort and similar nation-wide studies. We previously used this exposure index to assess associations between combined exposure and neonatal outcomes [23]. In this prior study, we found that greater prenatal exposure to combined environmental and social hazards was associated with lower birthweight and gestational age at birth as well as a high risk of preterm birth. We observed effect modification by pregnant person race, educational attainment, and urbanicity. Here we aimed to examine the distribution of our combined exposure index for the ECHO-wide Cohort and assess inequities in combined exposures during pregnancy as a function of population demographic and socioeconomic descriptors. Greater exposure to environmental hazards and social stressors may reflect the legacy of redlining and other policies that promote racial and ethnic segregation in the U.S. [24,25,26]. Harmful environmental exposures, unfavorable social conditions, structural racism, and poor maternal and child health outcomes may form a synergistic epidemic that disproportionately impacts marginalized communities [27]. Our goal was to document the methods used to develop the index and better understand how it may be used to examine health disparities within the ECHO-wide cohort.

## 2. Materials and Methods

### 2.1. Study Population

The NIH ECHO Program combines 69 ongoing pregnancy and pediatric studies from 31 cohorts across the U.S. into one ECHO-wide Cohort [28,29]. The goal of the ECHO-wide Cohort Study is to examine environmental factors associated with child health [30]. ECHO-wide Cohort data include a combination of extant study-specific data with prospective data collection using a common protocol across studies. The current analysis used previously collected or extant data to evaluate census tract-level social and environmental stressors in relation to demographic and socioeconomic measures during pregnancy. Individual study cohorts eligible for this analysis had 30 or more pregnancies with both residential history and demographic data between 2010 and 2019. Five cohorts recruited preterm or very-low-birthweight infants from neonatal intensive care units. Two cohorts recruited specific demographic groups (Black/African American and Puerto Rican). Six cohorts recruited participants with an autism spectrum disorder diagnosis or an older sibling with a diagnosis, and one cohort recruited pregnant people who smoked and refused cessation.

Because data are continuously uploaded to the ECHO platform, we used data from the 4 March 2022 data lock (Figure S1). Participant addresses were geocoded in ArcGIS Pro Streetmap Premium Geocoder. Approximately 87% of addresses had a high-quality match (point or specific street address), which was required for inclusion in this analysis. We assigned a census tract identifier to each participant address using the 2010 census tract boundaries.

In this analysis, we included all unique pregnancies with available data. Thus, a pregnant participant could contribute data from more than one pregnancy while enrolled in their original cohort study. All participants gave informed consent in their original cohort studies using approved methods. All participants included in these analyses provided additional consent to share data with the ECHO consortium. The ECHO-wide Cohort Data Collection Protocol was approved by either the ECHO single Institutional Review Board (IRB) or the original ECHO cohort’s local IRB.

### 2.2. Developing the Combined Exposure Index

We examined several existing local-, state-, and national-level indices to derive a list of candidate indicators of environmental hazards and social stressors [2,4,5,8,9,10,13]. Our goal was to identify as many indicators as possible that would reflect both chemical or physical environmental hazards (e.g., exposure to ambient air pollution) and social stressors (e.g., factors that correlated with perceived stress or measures, such as transportation and housing quality). After reviewing existing tools, we identified a list of candidate indicators that met two key criteria. First, indicators were eligible for inclusion if a publicly available data source with nationally representative data could be identified. Second, indicators were eligible if sufficient temporal data were available (e.g., at least every 3 years, with some exceptions noted in the following sections). Our final list of indicators was heavily influenced by two existing tools: CalEnviroScreen and the SVI. During our methods development process, we evaluated additional individual indicators related to the social determinants of health (e.g., crime and medically underserved areas) but found that data for other indicators of interest lacked either national or temporal coverage.

### 2.3. Data Sources

All of our indicators were obtained from publicly available data sources with national coverage (Table 1) [4,31,32,33,34,35,36]. Environmental indicators at the neighborhood level captured hazards in two broad classes: ambient air pollutants and features of the built environment. Environmental data were obtained from six sources: the National Land Cover Database (NLCD) [31], National Emissions Inventory (NEI) [32], National Priorities List (NPL) [33], Risk-Screening Environmental Indicators (RSEI) Model [34], EPA Fused Air Quality Surface Downscaling Files (FAQSD) for ambient air pollutants (fine particulate matter with an aerodynamic diameter less than 2.5 μm [PM_2.5_] and ozone [O_3_]) [35], and the National Highway Performance and Monitoring System (NHPMS) [36].

Social stressors were derived from the SVI [4]. These indicators represented a number of neighborhood-level factors that reflect SES and may influence population susceptibility. In our index, we included indicators of educational attainment (percentage of persons over the age of 25 without a high school diploma or the equivalent), employment status (unemployment percentage), per capita income, poverty (percentage of persons in poverty), age distribution (percentage of persons over the age of 65 and percentage of persons under the age of 18), disability (percentage of persons with a disability), household composition (percentage of single-parent households with children under the age of 18), race and ethnicity (percentage of persons of a race or ethnicity other than non-Hispanic White), language (percentage of persons who speak English “less than well”), and housing type (percentage of housing structures with 10+ units, percentage of mobile homes, percentage of overcrowded homes, percentage of households with no vehicle available, and percentage of persons in group quarters). The SVI includes the percentage of persons of a racial or ethnic group other than non-Hispanic White as a proxy for the “social and economic marginalization of certain racial and ethnic groups, including real estate discrimination” [37] in the United States. Demographic indicators at the census tract level are meant to further describe the contextual factors within a census tract rather than its composition [38]. These data were drawn from the U.S. Census Bureau American Community Survey (ACS) [39,40]. and were available at the census tract level.

### 2.4. Addressing Spatial and Temporal Alignment in the Environmental and Social Data Sets

Several environmental data sets were available at spatial resolutions other than the census tract level (Table 1). Therefore, we performed spatial aggregation analyses to assign values to each census tract in the United States. Data from the NLCD were available as raster files with a 30 m resolution. We aggregated these data to the census tract level using spatial overlay and extract functions, and we calculated the average percentage of land within the census tract with tree cover or land that was classified as an impervious surface. To ensure consistent interpretation of all indicators, the tree cover values were reversed (100%—% tree cover) to ensure that higher values were indicative of worse exposures (less tree cover). Images to measure tree cover are collected during the growing season and represent peak cover [41]. NEI and NPL sites were available as point estimates. We aggregated these as weighted sums of locations within the census tract and surrounding buffers around the census tract using weights specified by CalEnviroScreen 3.0 methodology [13]. The NHPMS estimates of annual average daily traffic (AADT) were available with line geography; census tract AADT was defined as the average number of vehicles per total area within the tract. RSEI and EPA FAQSD data were available at the census tract level, and no additional spatial manipulation was needed.

The combined exposure (CE) index was developed for each year of our study period (2010–2019) (Table 1). For data sets with less than annual temporal resolution, we imputed annual data from neighboring years to develop a data set with full temporal coverage. We elected to use the same exposure data for several years for two main reasons. First, we wanted to use methods that would be easily reproducible by other analysts. Second, we use long-term averages in our index, which are relatively stable over shorter periods of time (i.e., 5 years or fewer); therefore, more sophisticated methods of interpolation were not necessary. Because all of our data were collected prior to the COVID-19 pandemic, we assumed stable trends in our variables. NLCD data on impervious surfaces available for 2011, 2016, and 2019 were applied in annual exposure indices to cover 2010–2014, 2015–2017, and 2018–2019, respectively. For tree cover, 2011 and 2016 data were applied for the 2010–2014 and 2016–2019 indices, respectively. Data on NPL sites were taken from the 2017 list. Because the list has not changed substantially in recent history, we assumed that sites on the list as of 2017 were hazardous for the entire study period. RSEI data were available on an annual basis from 2010–2019. Data from 2010 through 2017 were based on RSEI model v237; 2018 data were based on model v238; and 2019 data from model v239. Data from the EPA FAQSD were available on an annual basis from 2010 through 2017; estimates from 2017 were used for 2018 and 2019. Annual AADT estimates were available for years 2011–2017; 2011 estimates were used for the 2010 index; and 2017 estimates were used in indices for 2018 and 2019.

Indicators of social stressors, which were originally developed by the Centers for Disease Control and Prevention (CDC), were acquired from data products labeled as 2010, 2014, 2016, and 2018, which were developed from 5-year estimates from the ACS from 2006–2010, 2010–2014, 2012–2016, and 2014–2018, respectively. Considering the temporal span of the ACS data sets, we applied these data, which represent 5-year averages, to our index based on the midpoint years of the data sets. Therefore, CDC data from 2006–2010 were applied to the 2010 index; data from 2010–2014 were applied to the index for years 2011–2012; data from 2012–2016 were applied to the index for years 2013–2015; and data from 2014–2018 were applied to the index for years 2016–2019.

### 2.5. Calculating Scores

We adopted methods used by CalEnviroScreen 3.0 (which was the most recent version of CalEnviroScreen at the time of data collection and methods development) to develop our national CE index [13]. We converted raw inputs for each variable (i.e., annual census tract values for each indicator) to percentiles scaled from 0 to 1.

We first calculated the environmental (ENV) and social (SOC) components of the CE index. To calculate ENV, the percentiles for each indicator were averaged to generate an air pollution subscore and a built environment subscore (Table 1). The final ENV index is the weighted average of the air pollution subscore (weight = 1) and the built environment subscore (weight = 0.5). The ENV scores were weighted to provided consistency with previous studies [3,10,13,23,42]. The built environment subscore (called “environmental effects” in the CalEnviroScreen methodology) is assigned half the weight of the ambient air pollution subscore due to the uncertainty in how these exposures are associated with health outcomes; a previous study of the CalEnviroScreen tool found that census tract rankings were robust to different weights [13,43]. Values for the ENV index range from 0 to 1. To calculate the SOC, percentiles of the inputs (Table 1) were averaged without weighting.

CE values were calculated as the product of the ENV and SOC scores (i.e., CE = ENV × SOC). This is the same approach used by CalEnviroScreen and other similar indices [2,3,42] and reflects the body of evidence that suggests an interaction between neighborhood factors (e.g., neighborhood SES) and environmental exposures (e.g., air pollution) on childhood health outcomes [44,45,46,47,48]. Values for the CE index range from 0 to 1, where higher scores represent higher levels of exposure to environmental hazards and social stressors.

### 2.6. Assigning Exposures

Participants were assigned a CE value based on the census tract in which they lived during gestation. Participants who had a pregnancy spanning more than one calendar year were assigned a CE value based on the year during which a greater proportion of the gestation occurred. For participants who moved during their pregnancy and who had residential history data (5%), we assigned CE values based on the census tract in which they lived for the greater proportion of the pregnancy.

### 2.7. Predictors of High Exposure

We were interested in differences in exposure based on characteristics such as race, ethnicity, and SES. Previous studies have suggested that race or ethnicity and SES may modify how environmental and social factors at the neighborhood level influence health outcomes [49,50,51,52,53]. Factors such as overt and covert racism, perceptions of social status, and social isolation may play a role in modifying the effects of neighborhood-level factors [52]. Pregnant people were characterized based on their self-reported race, ethnicity, and educational attainment (a proxy for individual-level SES). Because data on parental educational attainment were collected at multiple time points by several ECHO cohorts, we used a data source hierarchy to assign values to participants. Whenever available, we used data from the first prenatal visit. If prenatal data on parental educational attainment were not available, we used data from a childhood visit.

We were also interested in how exposures may differ for pregnant people in rural areas compared with urban areas and by geographic region. Previous studies have found that disparities in environmental exposures (e.g., air pollution and green space) by race and ethnicity differ by region or urbanicity [54,55,56]. Therefore, we also classified pregnant people as living in urban and rural regions using the 2013 Rural–Urban Continuum Code (RUCC) for the county in which they lived the longest during pregnancy [57]. We defined four primary regions of interest based on U.S. Census definitions: Northeast, South, Midwest, and West.

### 2.8. Statistical Analyses

We explored the distribution of the index values by year and national region for all census tracts included in our data set in two ways. First we included all census tracts and years. Then we limited our analysis to only tracts where pregnant ECHO participants resided during our study period (2010–2019). We used violin plots to examine the distribution of each of the component scores (ENV and SOC) and the CE index. We examined trends for the entire study area and specific regions in the U.S.

Our primary analysis tested the hypothesis that pregnant people from historically marginalized racial or ethnic groups and pregnant people with lower SES would be at greater risk of experiencing higher combined exposures during pregnancy. We also examined whether pregnant people in rural counties were at higher risk of living in a high-exposure census tract relative to pregnant people in urban counties. Analyses were conducted using data for the full ECHO cohort and by geographic region. Each characteristic of interest was analyzed separately; we did not mutually adjust for other characteristics (e.g., we did not include race and ethnicity in the same model).

High exposure was defined as a CE value greater than or equal to 0.5. This is the theoretical median for the CE index, which can each range from 0 to 1. In a secondary analysis, we defined high exposure as a CE value above the median of all observed values (all U.S. census tracts and years). In this secondary analysis, our threshold for high exposure was greater than or equal to 0.23 (the median of observed CE values for study participants). Because no rural census tracts had CE values above 0.5, our analysis of urban–rural disparities in exposure was limited to our secondary analysis.

We estimated the risk of being in a high-exposure census tract by race (Black or other racial groups, including Asian, Native Hawaiian and other Pacific Islander, American Indian or Alaska Native, and Multiple Races or Another Race vs. White), ethnicity (Hispanic vs. non-Hispanic), and educational attainment (less than high school or high school diploma/General Educational Development [GED] vs. some college and above). Although there are challenges for interpretation when using non-Hispanic White populations as the reference group to examine differences by race and ethnicity [58], we elected to use White as the reference group for the race category and non-Hispanic as the reference group for the ethnicity category here because participants who identified as White and non-Hispanic made up the largest proportions of our study population for those two demographic groups. We used Poisson regression with robust variance estimates [59] to calculate risk ratios for all participants at the national level and for models stratified by geographic region. We use the term relative risk to describe the likelihood of living in a high-exposure census tract relative to our reference populations.

To examine potential effect modification by geographic region, we examined the stratum-specific associations for each subgroup and included a product term of the participant characteristic (race, ethnicity, educational attainment, and urbanicity) and region to derive interaction *p*-values. We considered two-sided *p* < 0.10 as evidence for effect modification based on the *p*-value of the interaction terms. Because of sample size limitations, we did not include interaction terms when examining participants stratified by county type (urban vs. rural).

We included a sensitivity analysis to assess the potential influence of a residential move (defined as moving to a different census tract) during pregnancy on our results. In our study population, 640 (4.5%) pregnant people moved to a different census tract during their pregnancies. In this sensitivity analysis, we stratified our study population into two groups: those who moved during pregnancy and those who did not. We included an interaction term as the moving status and region in our sensitivity analysis.

## 3. Results

### 3.1. Temporal Trends and Regional Differences in the CE Index

**Figure 1:**
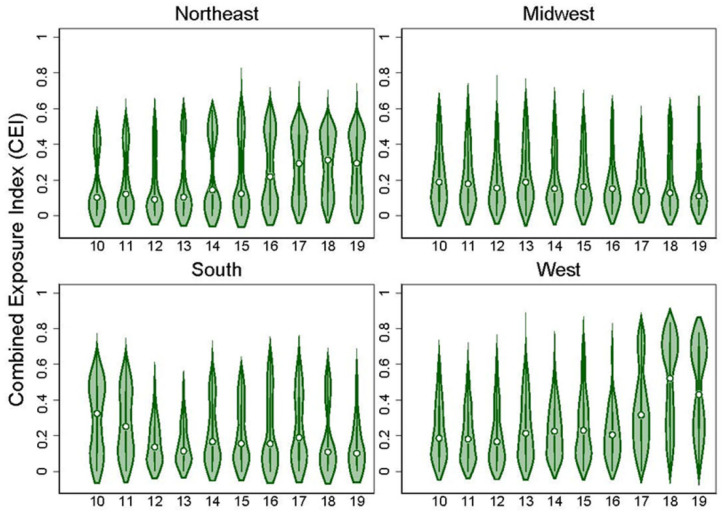
Violin plots showing the distribution of the combined exposure (CE) index for all census tracts that contained an ECHO participant by region for the years 2010–2019.

Across the U.S., CE values tended to be stable from year to year (Figure S2). Mean CE values tended to be higher in the West, but differences between regions were small (Figure S3, Table S1). When including only census tracts in which ECHO participants lived, exposures varied by year (Figure S4). Across all ECHO census tracts, exposures tended to be higher in later years (2017 onward) compared with earlier years. Combined exposures to environmental hazards and social stressors were higher in the West and Northeast regions of the country (Figure 1). Plots of the individual component scores (ENV and SOC) showed similar trends as the CE index (Figures S5–S12 in the Supplementary Materials).

### 3.2. Pregnancies Included in This Analysis (2010–2019)

Residential information and demographic variables were available for 14,072 pregnancies from 46 participating ECHO cohorts (Table 2, Figure S1). Of these pregnancies, 93% were pregnant people who contributed data to the study once. Our participants lived throughout the U.S. (Graphical Abstract; Figure S13) and represent 6264 different census tracts. Most participants lived in the Northeast region (*n* = 5642, 40%), followed by the West (*n* = 4012, 28%), Midwest (*n* = 2746, 20%), and South (*n* = 1672, 12%) regions. The majority of pregnant people included in our study identified as White (67%) and non-Hispanic (80%). The proportion of the study population who identified as a specific racial or ethnic group differed by region (Table 2). Participants who identified as Hispanic were more likely to live in the West (27%) and the Northeast (26%) compared with the Midwest (4%) and South (9%), and participants who identified as Black were more likely to live in the South (31%) compared with the Midwest (14%), Northeast (13%), and West (6%). Educational attainment among enrolled participants was high in our cohort, with 78% of pregnant people reporting at least some college (no degree) or higher education. Most participants (85%) lived in counties that were designated as metropolitan based on the 2013 RUCC classification system.

Table S2 in the Supplementary Materials details the number of participants from each cohort; cohorts contributed between 16 and 174 participants, with a median of 174 participants.

### 3.3. Relative Risk of Being in a “High” Exposure Census Tract by Participant Characteristics

Overall, pregnant people who identified as Black or another racial group (relative to White pregnant people) and pregnant people who identified as Hispanic (relative to non-Hispanic pregnant people) were at higher risk of living in a “high” exposure census tract where the CE index was higher than the theoretical median of 0.5 (Table 3). Differences in point estimates when examining geographic trends were evident (p-interaction = 0.016), although confidence intervals (CIs) for the stratified results were wide and overlapped. Racial differences in exposure were especially prevalent in the South, where Black pregnant people had a six times higher risk of being in a “high” CE exposure census tract than White pregnant people (RR = 6.04, 95% CI: 2.45–14.36). Risks for Black pregnant people were also elevated relative to White pregnant people in the Midwest region (RR = 5.21, 95% CI: 2.44–11.14). In contrast, among those residing in the Northeast, pregnant people who identified as members of other racial groups had the highest risks compared with White pregnant people (RR = 3.11, 95% CI: 2.20–4.40). For pregnant people identifying as Hispanic, the risk of living in a high-exposure tract was higher compared with non-Hispanic pregnant people overall (RR = 2.30, 95% CI: 1.77–3.00). Risks were similar for Hispanic pregnant people living in the Northeast (RR = 2.29, 95% CI: 1.58–3.31) and the West (RR = 2.91, 95% CI: 1.93–4.39) (p-interaction for ethnicity < 0.001).

We also observed socioeconomic inequalities in high exposure risk (Table 3). Pregnant people with less than a high school education had higher risks of living in high CE census tracts compared with pregnant people with some college education and above (RR = 2.27, 95% CI: 1.66–3.10). Risks for this group were similar in the Northeast, Midwest, and South, but not in the West. Similar trends were observed for pregnant people with a high school degree relative to those with some college and above.

### 3.4. Results When Defining “High” Census Tracts as above the Observed Median for the CE Index (0.23)

When defining “high” exposure as the median of all observed values (≥0.23), the patterns we observed were similar, although the relative risks of being in a high-exposure census tract during pregnancy were attenuated (Table S3). Relative to White pregnant people, Black pregnant people had a higher risk of being in a high-exposure tract (RR = 2.33, 95% CI: 1.93–2.82), with pregnant people living in the Midwest experiencing the highest relative risk (RR = 3.65, 95% CI: 2.49–5.35) (p-interaction < 0.001). Hispanic pregnant people had a higher risk of living in a high-exposure census tract relative to non-Hispanic pregnant people both nationally (RR = 1.62, 95% CI: 1.43–1.84) and in each of the regions identified (except the South), although the interaction term was no longer significant when using this alternative definition of “high” exposure (p-interaction = 0.743). Trends for pregnant people with lower educational attainment were also consistent with the previous analysis, where pregnant people with lower reported educational attainment were at higher risk of living in a high-exposure census tract.

We observed that pregnant people living in metro counties had a higher risk of living in a census tract with a CE value ≥ 0.23 (RR = 3.03, 95% CI: 1.75–5.27) relative to those living in non-metro counties (Table S3). Relative risks were highest for pregnant people living in metro counties in the West (RR = 12.68, 95% CI: 5.57–28.90) and lowest for pregnant people living in metro counties in the South (RR = 1.28, 95% CI: 0.74–2.22), although CIs tended to be wide. Estimates of this risk were not available for participants in the Northeast due to sample size limitations.

### 3.5. Results of the Sensitivity Analysis Considering Movers and Non-Movers

The results were generally not sensitive to whether pregnant people moved during their pregnancy (Table S4). When stratifying participants by race or ethnicity, the risks for pregnant people who moved during pregnancy were somewhat lower compared with pregnant people who did not move. However, when stratifying by educational attainment (our proxy for SES), there were differences between pregnant people who moved and who did not move. Movers in the lower educational attainment group experienced a slightly higher risk of being in a high-exposure census tract (based on the theoretical median value of 0.5) relative to movers in the higher educational attainment group, although CIs were wide and overlapped.

## 4. Discussion

To facilitate studies of combined effects from multiple environmental and social stressors on childhood health outcomes, we leveraged existing national data sets to develop a combined exposure index. Within the nationwide ECHO-wide Cohort, we assigned CE index scores based on residence and timing of pregnancy and assessed differences in exposure by key demographic and socioeconomic groups. Overall, our results show that pregnant people from minoritized racial and ethnic groups and pregnant people with lower educational attainment may be at greater risk of living in a high-exposure census tract. These trends were similar whether we defined “high” exposure as above the theoretical median value (0.5) for the index or the observed median value (0.23), although the magnitude of risk was greater when using the theoretical median. Because we did not mutually adjust for other demographic characteristics, these results should be interpreted with caution. However, our results are consistent with several recent studies that have documented disparities in exposure to social and environmental risk factors by minoritized racial and ethnic groups, including air pollutants [60,61,62], parks and green space [56,63], and Superfund sites [64,65]. These differences in combined exposures to environmental hazards and social stressors likely reflect the legacy of structural and systemic racism that persists today in the U.S. [66,67].

Our national-level CE index may be useful in several future research contexts. We previously applied these measures to a study of combined exposures during the prenatal period and perinatal outcomes, including gestational age, preterm birth, and small- and large-for-gestational age, and observed that higher index scores were associated with decreased gestational age at birth [23]. However, our use of a combined exposure index, which collapses exposure information into a single metric, does not allow us to differentiate between exposures to identify the most important component or components of the mixture. A better understanding of which exposures drive associations with health outcomes is needed to elucidate the mechanisms underlying observed differences in exposure and in health effects. Similarly, identifying key components within the mixture will help identify policy and program options for addressing them. Future work may aim to investigate outcomes along the pathway between combined neighborhood exposures and perinatal outcomes, such as psychosocial stress or oxidative stress [22]. Additionally, we have estimates of each indicator for each year of the index to ensure that exposures could be investigated as separate predictors. To further elucidate how these exposures interact to influence child health outcomes, statistical methods for mixtures that leverage machine learning, such as Bayesian Kernel Machine Regression or quantile-based g-computation, can be applied to the data set [68,69,70,71].

### Strengths, Limitations, and Future Directions

Our work benefits from several strengths. We were able to combine time-varying neighborhood-level exposures in two domains into a single index, which overcomes some of the limitations of other national-level tools. Additionally, we were able to assess exposures at the census tract level. The use of census tracts allowed us to capture some of the intra-county variability in exposures that may better capture the relationships between environment and health [72]. This dataset will be made available for ECHO and non-ECHO researchers to explore associations between combined exposures and other childhood health outcomes.

There are several sources of uncertainty to acknowledge when developing and interpreting the CE index. Many of these sources of uncertainty are common among other exposure indices in the literature [2,4,13]. First, the specific exposures of interest are not always clear for many built environment features. For example, living in close proximity to a Superfund site (i.e., a heavily polluted location that has been identified by the US EPA as hazardous to health and has been listed on the National Priorities List) has been linked to shortened life expectancy, particularly in areas with higher sociodemographic disadvantage [73]. However, the health risks presented by a specific site depend on a number of factors, including the historical activity at the site, groundwater and surface water conditions, and current land use practices. Thus, the relationships between proximity to Superfund sites and health outcomes may vary by location. Second, it is difficult to separate the effects of chemical and physical hazards associated with certain types of neighborhood environmental hazards (e.g., air pollutants and noise) from the effects of psychosocial stressors [74,75,76]. Third, it is challenging to empirically derive weights for component scores within the index. Whenever possible, we used CalEnviroScreen 3.0 (the version of CalEnviroScreen available at the time of methods development) weights for consistency [13].

Additionally, our work has other limitations to note. Similar to other national-level indices [2], we are limited by the availability of data sets; not all relevant indicators are included in this index. For example, we do not have indicators of water quality. Nationally representative data sets that account for both community water systems and private water systems are lacking. Thus, we cannot include indicators for hazards such as nitrates in drinking water that show clear sociodemographic patterns in the U.S. [77]. We are also not able to include indicators that may be more relevant in rural parts of the country, including pesticides and emissions from oil and gas operations. Our reliance on publicly available data sets precludes us from evaluating the entire U.S.; data are not routinely available for Alaska and Puerto Rico. Additionally, because of temporal limitations in existing data sets, for some indices, we applied the same data set to several years of the index. Contrasts in exposure were likely reduced due to this lack of temporally resolved data. Importantly, because of smaller sample sizes, we were not able to explore differences in exposure for other racial groups, including Asian, Native Hawaiian and other Pacific Islander, American Indian, and Alaska Native, and individuals reporting more than one race or other races. Previous work has demonstrated that exposures to environmental hazards and the incidence of adverse birth outcomes are higher among Asian, Native Hawaiian, and Pacific Islander populations [78,79,80] and American Indian or Alaska Native populations [81,82]. When applying our index to understand trends in exposure, we cannot rule out the influence of residential selection bias on our results [83], where individuals with higher SES are more able to choose desirable neighborhoods relative to individuals with lower SES. Lastly, our results are consistent with those from other studies in the United States but may not be generalizable outside of the country. In other regions of the world, there are likely important differences in the racial, ethnic, socioeconomic, and geographic distribution of environmental and social hazards.

## 5. Conclusions

To overcome some of the limitations of existing exposure indices and to facilitate health studies for the ECHO-wide Cohort, we developed a combined exposure index that accounts for environmental hazards and social stressors at the census tract level. We demonstrated the utility of our index by assessing differences in the risk of living in a high-exposure census tract for pregnant people from minoritized racial and ethnic groups, pregnant people with lower educational attainment, and pregnant people in urbanized counties. These exposure data may be useful in future studies on how neighborhood contexts influence health across childhood. Future work would benefit from national data sets for key environmental health concerns, such as water contaminants and pesticides, and social stressors that may have disproportionate effects, particularly in rural areas. Data collection efforts should focus on existing geographical gaps, including for Alaska, Hawaii, and Puerto Rico. These data sets are a requirement for capturing the full range of environmental hazards and social stressors that influence maternal and child health outcomes.

## Figures and Tables

**Table 1:** Indicators included in the combined exposure index.

Component Score	Source	Variable	Original Geography	Spatial Method	Temporal Resolution	Temporal Coverage	Final Indicator
ENV-BE	National Land Cover Database (NCLD)	Tree cover	Raster grid	Extract	2–3-year avg estimate	2011	% land area that is tree cover
ENV-BE	National Land Cover Database (NCLD)	Impervious surfaces	Raster grid	Extract	2–3-year avg estimate	2011	% land area that is impervious
ENV-BE	National Emissions Inventory (NEI)	Major emissions facilities	Point	Weighted count in buffers	Annual	1990, 1996–2002, 2005, 2008, 2011, 2014	Weighted sum of major emissions facilities within or near the census tract
ENV-BE	National Priorities List (NPL)	Superfund sites	Point	Weighted count in buffers	Annual	2015	Weighted sum of NPL sites within or near the census tract
ENV-AAP	Risk-Screening Environmental Indicators (RSEI) Model	Toxic air emissions	Census tract	N/A	Annual	2000–2017	Average toxicity-weighted concentration
ENV-AAP	National Highway Performance and Monitoring System (NHPMS)	Average annual daily traffic	Line	Overlay	Annual	2011–2017	Average vehicles per total area
ENV-AAP	EPA data fusion product	PM_2.5_ and ozone	Census tract	N/A	Daily	2002–2017	Average annual concentration (μg/m^3^, ppb)
SOC	American Community Survey (ACS) 5-year estimates	Education Employment Income Older adults Children Disability HH composition Minority status Language Housing types	Census tracts	N/A	5-year avg	2010–2016	% persons below poverty % unemployment ^1^ Per capita income% persons with no HS diploma ^2^ % persons aged 65 years and older % persons aged 17 years and younger % persons with a disability% single-parent HH with children < 18 years of age % persons who identify as a race or ethnicity other than non-Hispanic White % persons who speak English “less than well” % housing structures with 10+ units ^3^ % mobile homes ^3^ % overcrowded homes ^4^ % HH with no vehicle available % persons in group quarters

^1^ The unemployment rate is calculated by the ACS as the estimate of persons unemployed out of the total civilian population aged 16+ in the labor force. ^2^ Calculated using the population aged 25 and older. ^3^ Housing variables calculated as a percentage of the total estimated housing units. ^4^ Percentage of occupied housing units with more people than rooms. ACS, American Community Survey; avg, average; ENV-AAP, environmental component score (ambient air pollution subscore); ENV-BE, environmental component score (built environment subscore); HH, households; HS, high school; SOC, social component score.

**Table 2:** Demographics of ECHO children with residential history data (N = 14,072) by geographic region.

Characteristic	Full Cohort ^a^	Region 1 Northeast	Region 2 Midwest	Region 3 South	Region 4 West
N	14,072	5642	2746	1672	4012
Age at delivery, years, mean (SD)	30.6 (5.5) (*n* = 13,979)	31.1 (5.5) (*n* = 5584)	29.4 (5.3) (*n* = 2734)	30.4 (5.8) (*n* = 1656)	31.0 (5.4) (*n* = 4005)
Race, *n* (%)					
White	9031 (67%)	3271 (63%)	2041 (75%)	961 (59%)	2758 (71%)
Black	1802 (13%)	695 (13%)	376 (14%)	508 (31%)	223 (6%)
Other Race ^b^	2619 (19%)	1256 (24%)	313 (11%)	173 (11%)	877 (23%)
Missing	620	420	16	30	154
Ethnicity, *n* (%)					
Hispanic	2719 (20%)	1375 (26%)	121 (4%)	152 (9%)	1071 (27%)
Non-Hispanic	10,987 (80%)	3965 (74%)	2589 (96%)	1511 (91%)	2922 (73%)
Missing	366	302	36	9	19
Marital status, *n* (%)					
Married or living with a partner	7862 (80%)	3393 (78%)	1282 (84%)	681 (65%)	2506 (87%)
Widowed, separated, or divorced	373 (4%)	285 (7%)	26 (2%)	16 (2%)	46 (2%)
Single, never married, or partnered but not living together	1566 (16%)	665 (15%)	216 (14%)	352 (34%)	333 (12%)
Missing	4271	1299	1222	623	1127
Educational level, *n* (%)					
Less than high school	985 (7%)	440 (9%)	191 (7%)	126 (8%)	228 (6%)
High school degree, GED or equivalent	2007 (15%)	851 (17%)	396 (15%)	334 (20%)	426 (11%)
Some college, no degree, and above	10,412 (78%)	3793 (75%)	2101 (78%)	1198 (72%)	3320 (84%)
Missing	668	558	58	14	38
County type, *n* (%)					
Non-metro (RUCC 4–9)	2175 (15%)	1632 (29%)	433 (16%)	60 (4%)	50 (1%)
Metro (RUCC 1–3)	11,897 (85%)	4010 (71%)	2313 (84%)	1612 (96%)	3962 (99%)
Year of pregnancy, *n* (%)					
2010	299 (2%)	59 (1%)	77 (3%)	75 (4%)	88 (2%)
2011	1482 (11%)	456 (8%)	327 (12%)	163 (10%)	536 (13%)
2012	1513 (11%)	495 (9%)	367 (13%)	158 (9%)	493 (12%)
2013	1365 (10%)	402 (7%)	255 (9%)	238 (14%)	470 (12%)
2014	1508 (11%)	427 (8%)	326 (12%)	193 (12%)	562 (14%)
2015	1450 (10%)	330 (6%)	284 (10%)	271 (16%)	565 (14%)
2016	1195 (8%)	328 (6%)	166 (6%)	231 (14%)	470 (12%)
2017	1349 (10%)	737 (13%)	78 (3%)	145 (9%)	389 (10%)
2018	1730 (12%)	1046 (19%)	293 (11%)	126 (8%)	265 (7%)
2019	2181 (15%)	1362 (24%)	573 (21%)	72 (4%)	174 (4%)

^a^ The full cohort included 14,072 children who were born to 13,057 pregnant people. Overall, 93% of pregnant people were included in the study only once. ^b^ This group included participants who identified as Asian (*n* = 775, 6%), Native Hawaiian or other Pacific Islander (*n* = 71, <1%), American Indian or Alaska Native (*n* = 241, <1%) or Multiple Races or Another Race (*n* = 1532, 11%). ECHO, Environmental influences on Child Health Outcomes; GED, General Educational Development; RUCC, 2013 Rural–Urban Continuum Code; SD, standard deviation.

**Table 3:** Relative risks (95% CI) of living in a high-exposure census tract (defined as a CE index score ≥ 0.5) by maternal characteristics and geographic region (*n* = 14,072); *p*-values represent the *p*-value of the interaction term between the participant characteristic and region.

Characteristic	Full Cohort	Region 1 Northeast	Region 2 Midwest	Region 3 South	Region 4 West
Race *p* = 0.016					
White	Reference	Reference	Reference	Reference	Reference
Black	3.71 (2.14, 6.43)	4.35 (3.05, 6.20)	5.21 (2.44, 11.14)	6.04 (2.54, 14.36)	1.63 (1.02, 2.59)
Other	2.14 (1.38, 3.32)	3.11 (2.20, 4.40)	1.62 (1.16, 2.25)	2.08 (1.11, 3.87)	1.09 (0.87, 1.36)
Ethnicity *p* < 0.001					
Non-Hispanic	Reference	Reference	Reference	Reference	Reference
Hispanic	2.30 (1.77, 3.00)	2.29 (1.58, 3.31)	1.13 (0.76, 1.69)	0.38 (0.18, 0.79)	2.91 (1.93, 4.39)
Education *p* = 0.688					
Some college, no degree, and above	Reference	Reference	Reference	Reference	Reference
High school degree, GED or equivalent	2.09 (1.63, 2.67)	2.11 (1.72, 2.60)	2.33 (1.48, 3.67)	1.49 (0.82, 2.72)	1.73 (0.85, 3.52)
Less than high school	2.27 (1.66, 3.10)	2.13 (1.61, 2.81)	2.00 (1.30, 3.09)	2.25 (1.48, 3.43)	2.13 (0.86, 5.32)
County type					
Non-Metro	Reference	Reference	Reference	Reference	Reference
Metro	7.09 (1.97, 25.55)	N/A	N/A	3.42 (1.06, 11.05)	N/A

CE, combined exposure index, CI, confidence interval; GED, General Educational Development.

## Data Availability

De-identified data from the ECHO Program are available through NICHD’s Data and Specimen Hub (DASH). DASH is a centralized resource that allows researchers to access data from various studies via a controlled-access mechanism. Researchers can now request access to these data by creating a DASH account and submitting a Data Request Form. The NICHD DASH Data Access Committee will review the request and provide a response in approximately two to three weeks. Once granted access, researchers will be able to use the data for three years. See the DASH Tutorial for more detailed information on the process.

## Supplementary Materials

The following supporting information can be downloaded at https://www.mdpi.com/article/10.3390/ijerph20146339/s1, Table S1: Summary (mean and SD) of the Environmental Exposure Index, Social Exposure Index, and Combined Exposure Index by year and geographic region for all National Census Tracts. Table S2: Summary of ECHO cohorts included in this study. Table S3: Relative risks (95% CI) of living in a high-exposure census tract (defined as a combined exposure index score ≥ 0.23) by maternal characteristics and geographic region (N = 14,072). Table S4: Relative risks (95% CI) of living in a high-exposure census tract by maternal characteristics and geographic region: sensitivity analysis stratified by mothers who moved vs. did not move during pregnancy (N = 14,072). Figure S1: Flowchart outlining the inclusion of study participants in the analytic cohort. Figure S2: Violin plots showing the distribution of the combined exposure index for the United States for the years 2010–2019. Figure S3: Violin plots showing the distribution of the combined exposure index for the United States by region for the years 2010–2019. Figure S4: Violin plots showing the distribution of the combined exposure index for all census tracts that contained an ECHO participant for the years 2010–2019. Figure S5: Violin plots showing the distribution of the environmental component score for the United States for the years 2010–2019. Figure S6: Violin plots showing the distribution of the environmental component score for the United States by region for the years 2010–2019. Figure S7: Violin plots showing the distribution of the environmental component score for all census tracts in which ECHO participants lived for the years 2010–2019. Figure S8: Violin plots showing the distribution of the environmental component score for all census tracts in which ECHO participants lived by region for the years 2010–2019. Figure S9: Violin plots showing the distribution of the social component score for the United States for the years 2010–2019. Figure S10: Violin plots showing the distribution of the social component score for the United States by region for the years 2010–2019. Figure S11: Violin plots showing the distribution of the social component score for all census tracts in which ECHO participants lived for the years 2010–2019. Figure S12: Violin plots showing the distribution of the social component score for all census tracts in which ECHO participants lived by region for the years 2010–2019. Figure S13: The number of participants by state and region used to assess geographic differences in the combined exposure index.

## Abbreviations

AADT, annual average daily traffic; ACS, American Community Survey; ATSDR, Agency for Toxic Substances and Disease Registry; CDC, Centers for Disease Control and Prevention; CE, combined exposure index; CI, confidence intervals; ECHO, Environmental Influences on Child Health Outcomes; EJI, Environmental Justice Index; EPA, Environmental Protection Agency; ENV, environmental; FAQSD, Fused Air Quality Surface Downscaling Files; GED, General Educational Development; IRB, Institutional review board; NEI, National Emissions Inventory; NHPMS, National Highway Performance and Monitoring System; NIH, National Institutes of Health; NPL, National Priorities List; SES, socioeconomic status; SOC, social; SVI, Social Vulnerability Index; NLCD, National Land Cover Database; O3, ozone; RSEI, Risk-Screening Environmental Indicators; RUCC, Rural–Urban Continuum Code; U.S., United States.
