# Supplementary material for: Developing a National-Scale Exposure Index for Combined Environmental Hazards and Social Stressors and Applications to the Environmental Influences on Child Health Outcomes (ECHO) Cohort
Source: Int J Environ Res Public Health. 2023 Jul 10;20(14):6339. doi: 10.3390/ijerph20146339 (PMC10379099; doi:10.3390/ijerph20146339)
Supplement: Supplementary file 1 [file ijerph-20-06339-s001.zip › ijerph-2446061-supplementary.pdf]

SUPPLEMENTARY MATERIALS

Supplementary Tables (S1-S4)

Supplementary Figures (S1-S13)

Table S1 Summary (mean and SD) of the Environmental Exposure Index, Social Exposure Index, and Combined Exposure Index by year and geographic region for all National Census Tracts.

Index	Year	National	Region 1 Northeast	Region 2 Midwest	Region 3 South	Region 4 West
Environmental Exposure Index (ENV)	2010	0.45 (0.14)	0.45 (0.15)	0.48 (0.14)	0.44 (0.13)	0.43 (0.15)
	2011	0.45 (0.14)	0.44 (0.15)	0.47 (0.14)	0.44 (0.13)	0.45 (0.16)
	2012	0.45 (0.15)	0.44 (0.15)	0.50 (0.14)	0.42 (0.14)	0.45 (0.16)
	2013	0.45 (0.15)	0.45 (0.14)	0.49 (0.13)	0.40 (0.14)	0.48 (0.16)
	2014	0.45 (0.15)	0.43 (0.15)	0.46 (0.15)	0.42 (0.13)	0.49 (0.18)
	2015	0.45 (0.16)	0.45 (0.15)	0.47 (0.15)	0.41 (0.14)	0.50 (0.18)
	2016	0.45 (0.16)	0.44 (0.15)	0.45 (0.15)	0.43 (0.13)	0.50 (0.19)
	2017	0.45 (0.16)	0.41 (0.15)	0.44 (0.15)	0.42 (0.14)	0.53 (0.18)
	2018	0.45 (0.16)	0.41 (0.15)	0.44 (0.15)	0.42 (0.14)	0.53 (0.18)
	2019	0.45 (0.16)	0.42 (0.15)	0.44 (0.15)	0.43 (0.14)	0.52 (0.17)
Social Exposure Index (SOC)	2010	0.50 (0.29)	0.46 (0.29)	0.46 (0.27)	0.53 (0.29)	0.53 (0.30)
	2011	0.50 (0.29)	0.45 (0.29)	0.45 (0.28)	0.54 (0.28)	0.53 (0.29)
	2012	0.50 (0.29)	0.45 (0.29)	0.45 (0.28)	0.54 (0.28)	0.53 (0.29)
	2013	0.50 (0.29)	0.45 (0.29)	0.45 (0.28)	0.54 (0.28)	0.53 (0.29)
	2014	0.50 (0.29)	0.45 (0.29)	0.45 (0.28)	0.54 (0.28)	0.53 (0.29)
	2015	0.50 (0.29)	0.45 (0.29)	0.45 (0.28)	0.54 (0.28)	0.53 (0.29)
	2016	0.50 (0.29)	0.45 (0.29)	0.45 (0.28)	0.54 (0.28)	0.53 (0.29)
	2017	0.50 (0.29)	0.45 (0.29)	0.45 (0.28)	0.54 (0.28)	0.53 (0.29)
	2018	0.50 (0.29)	0.45 (0.29)	0.45 (0.28)	0.54 (0.28)	0.53 (0.29)
	2019	0.50 (0.29)	0.45 (0.29)	0.45 (0.28)	0.54 (0.28)	0.53 (0.29)
Combined Exposure Index (CEI)	2010	0.23 (0.17)	0.22 (0.18)	0.23 (0.17)	0.24 (0.15)	0.24 (0.18)
	2011	0.23 (0.17)	0.21 (0.18)	0.22 (0.17)	0.24 (0.16)	0.25 (0.18)
	2012	0.23 (0.17)	0.21 (0.17)	0.24 (0.18)	0.23 (0.15)	0.25 (0.18)
	2013	0.23 (0.17)	0.22 (0.18)	0.23 (0.17)	0.22 (0.15)	0.27 (0.19)
	2014	0.23 (0.17)	0.21 (0.17)	0.22 (0.17)	0.23 (0.15)	0.28 (0.21)
	2015	0.23 (0.18)	0.22 (0.18)	0.22 (0.17)	0.22 (0.15)	0.28 (0.21)
	2016	0.23 (0.18)	0.21 (0.17)	0.21 (0.17)	0.23 (0.15)	0.27 (0.21)
	2017	0.23 (0.18)	0.20 (0.17)	0.21 (0.17)	0.23 (0.15)	0.29 (0.21)
	2018	0.23 (0.18)	0.20 (0.17)	0.21 (0.17)	0.23 (0.15)	0.29 (0.21)
	2019	0.23 (0.18)	0.20 (0.17)	0.21 (0.17)	0.23 (0.16)	0.29 (0.20)

Table S2 Summary of ECHO cohorts included in this study.

Cohort name	Sample size in this study (03/04 lock)	Special population
Prematurity and Respiratory Outcomes Program (DINE-PROP)	175	Preterm infants
Trial of Late Surfactant	40	Preterm infants
NICU Hospital Exposures and Long-Term Health (DINE-NICUHEALTH)	63	Very-low-birthweight infants
Preterm Erythropoietin Neuroprotection Trial (DINE-PENUT)	57	Newborn intensive care unit (NICU) infants
43rd Multicenter Airway Research Collaboration	173	
Healthy Start	751	
BAMBAM	337	
MINNIE	402	
Boricua Youth Study	85	Puerto Rican youth
Atlanta ECHO Cohort of Emory University	243	Self-identified as Black/African American
Safe Passage Study	1191	
(PETALS) Pregnancy Environment and Lifestyle Study	690	
(KPRB) Kaiser Permanente Research Bank	471	
Microbes, Allergy, Asthma & Pets Study (MAAP)	22	
Wisconsin Infant Study Cohort (WISC)	105	
Infant Susceptibility to Pulmonary Infections and Asthma Following RSV Exposure (INSPIRE)	182	
MADRES	279	
ReCHARGE: Revising the CHARGE Study children	103	Autism spectrum disorder (ASD) diagnosis
Pittsburgh Girls Study	88	
New Hampshire Birth Cohort Study	1718	Rural cohort 18-45 years of age at enrollment. Participants were not planning to move, had fluency in English, and were living in a household with a private water supply (original but not current criterion).
CANDLE	207	
GAPPS	113	
ECHO-NOVI	451	Preterm infants
VDAART	249	
Vitamin C to Decrease Effects of Smoking in Pregnancy on Infant Lung Function	172	
In-Utero Smoke, Vitamin C, and Newborn Lung Function	23	Current cigarette smokers

Kennedy Krieger - BRSC	16	Older sibling with ASD
University of California Davis - BRSC	26	Older sibling with ASD
University of California - MARBLES	21	Older sibling with ASD
Infant Brain Imaging Study (IBIS)	34	Older sibling with ASD
Early Autism Risk Longitudinal Investigation (EARLI)	50	Older sibling with ASD
Rochester-Magee	122	
Magee	110	
Magee	368	
MARCH	519	
Illinois Kids Development Study	89	
Chemicals in our Bodies	202	
Generation NYC 2035	1597	
Programming of Intergenerational Stress Mechanisms	700	
First 100 Days Inova Childhood Longitudinal Study	694	
TIDES	495	
National Children’s Study IVS Salt Lake County (NCS-IVS)	76	
Alternate Recruitment Strategy Cache County (NCS-ARS)	213	
Home Observation of Periconceptional Exposures Utah (HOPE-Utah)	96	
Nulliparous Pregnancy Outcomes Study: Monitoring Mothers to be (NuMoM2B-Utah)	209	
Baby Affect & Behavior Study (BABY-Utah)	45	
ECHO-wide Study Cohort	14072	

Table S3 Relative risks (95% CI) of living in a high exposure census tract (defined as a combined exposure index score ≥ 0.23) by maternal characteristics and geographic region (N = 14,072). p-values represent the p-value of the interaction term between the participant characteristic and region.

**Characteristic**	**Full cohort**	**Region 1 Northeast**	**Region 2 Midwest**	**Region 3 South**	**Region 4 West**
Race $p < 0.001$					
White	Reference	Reference	Reference	Reference	Reference
Black	2.33 (1.93, 2.82)	2.20 (1.84, 2.62)	3.65 (2.49, 5.35)	2.50 (2.00, 3.13)	1.64 (1.38, 1.96)
Other Race	1.64 (1.40, 1.92)	1.81 (1.61, 2.03)	2.51 (1.91, 3.29)	1.56 (1.42, 1.72)	1.15 (1.04, 1.26)
Ethnicity $p = 0.743$					
Non-Hispanic	Reference	Reference	Reference	Reference	Reference
Hispanic	1.62 (1.43, 1.84)	1.66 (1.40, 1.97)	1.56 (1.17, 2.08)	1.54 (0.98, 2.43)	1.62 (1.38, 1.89)
Education $p = 0.194$					
Some college, no degree and above	Reference	Reference	Reference	Reference	Reference
High school degree, GED or equivalent	1.59 (1.42, 1.79)	1.55 (1.36, 1.77)	1.86 (1.49, 2.32)	1.66 (1.15, 2.41)	1.37 (1.05, 1.79)
Less than high school	1.81 (1.56, 2.10)	1.64 (1.43, 1.89)	2.20 (1.80, 2.69)	2.01 (1.21, 3.34)	1.67 (1.16, 2.40)
County type					
Non-Metro	Reference	Reference	Reference	Reference	Reference
Metro	3.03 (1.75, 5.27)	N/A	5.19 (1.87, 14.40)	1.28 (0.74, 2.22)	12.68 (5.57, 28.90)

GED, General Educational Development.

Table S4 Relative risks (95% CI) of living in a high exposure census tract by maternal characteristics and geographic region: sensitivity analysis stratified by mothers who moved vs. did not move during pregnancy (N = 14,072). p-values represent the p-value for the interaction between the participant characteristics and moving status on the odds of living in a high exposure census tract.

Characteristic	Full cohort	Did not move (n = 12,115)	Moved (n = 640)
Race<br>p-value = 0.082			
White	Reference (1)	Reference (1)	Reference (1)
Black	3.71 (2.14, 6.43)	3.79 (2.22, 6.45)	3.05 (1.42, 6.59)
Other Race	2.14 (1.38, 3.32)	2.18 (1.42, 3.34)	1.67 (0.84, 3.32)
Ethnicity<br>p-value = 0.166			
Non-Hispanic	Reference (1)	Reference (1)	Reference (1)
Hispanic	2.30 (1.77, 3.00)	2.34 (1.80, 3.04)	1.99 (1.30, 3.07)
Education<br>p-value = 0.464			
Some college, no degree, and above	Reference (1)	Reference (1)	Reference (1)
High school degree, GED or equivalent	2.09 (1.63, 2.67)	2.03 (1.57, 2.62)	3.28 (1.93, 5.60)
Less than high school	2.27 (1.66, 3.10)	2.24 (1.65, 3.05)	2.99 (1.45, 6.16)
County type			
Non-Metro	Reference (1)	Reference (1)	Reference (1)
Metro	7.09 (1.97, 25.55)	6.65 (1.93, 22.95)	N/A

GED, General Educational Development.

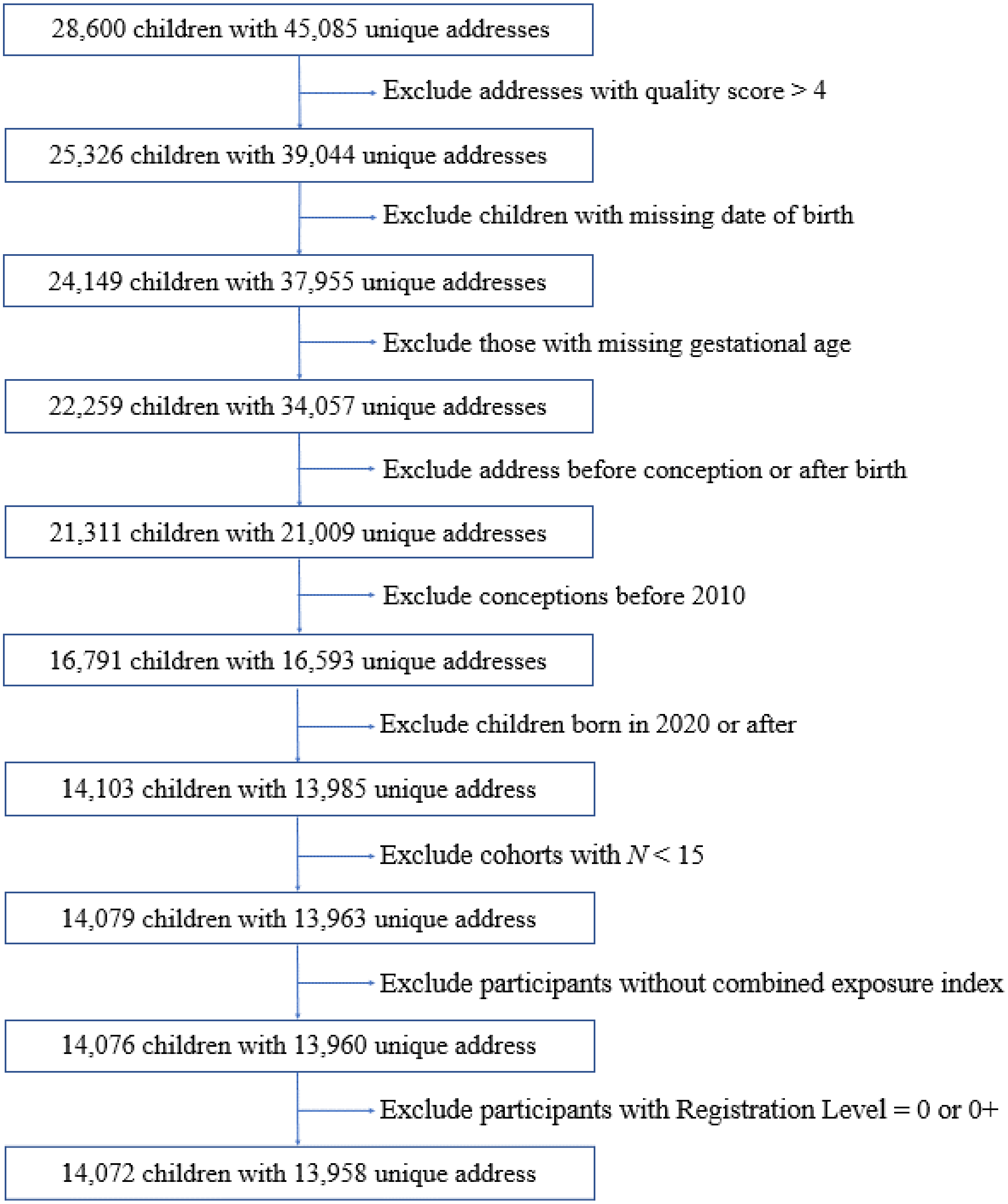

Figure S1. Flowchart outlining the inclusion of study participants in the analytic cohort.

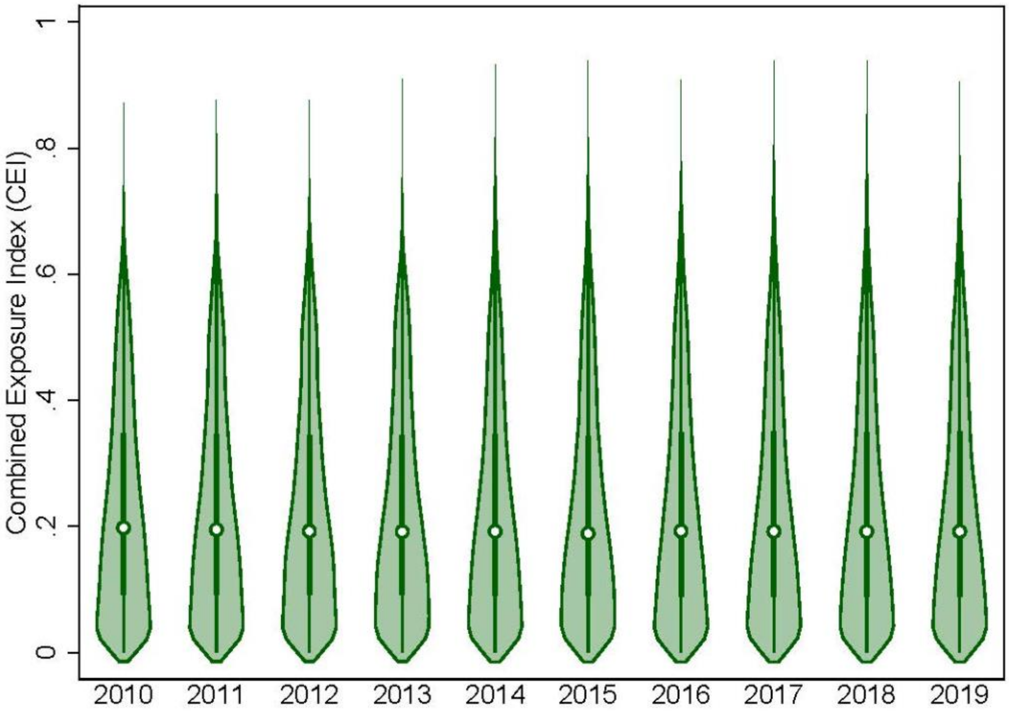

Figure S2. Violin plots showing the distribution of the combined exposure index for the United States for the years 2010-2019.

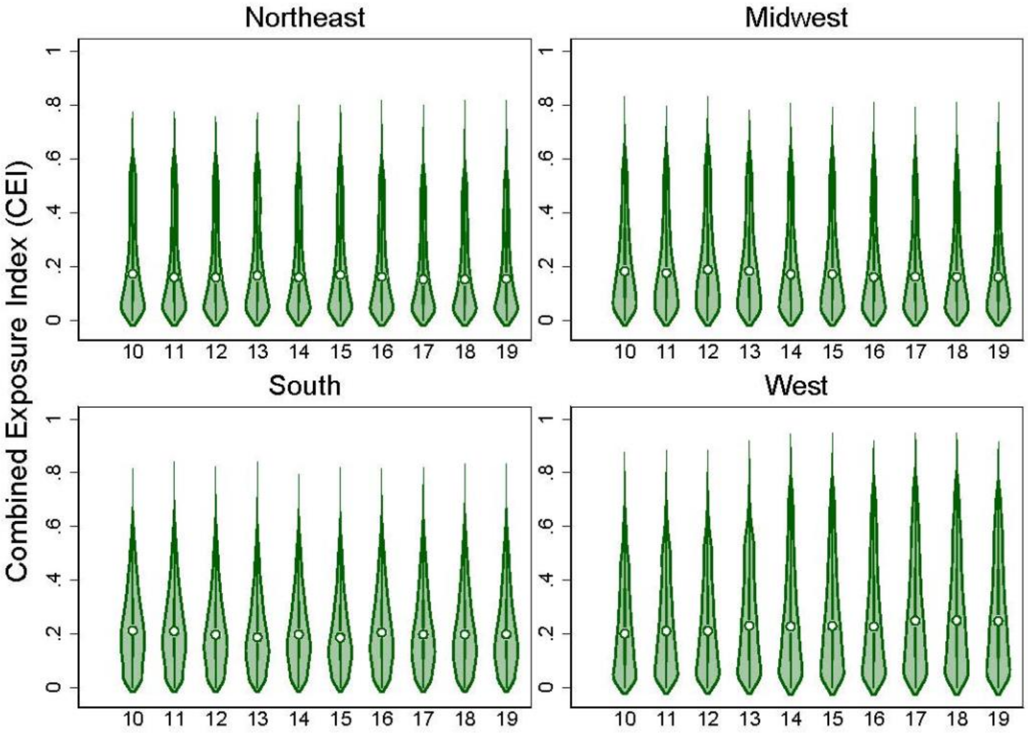

Figure S3. Violin plots showing the distribution of the combined exposure index for the United States by region for the years 2010-2019.

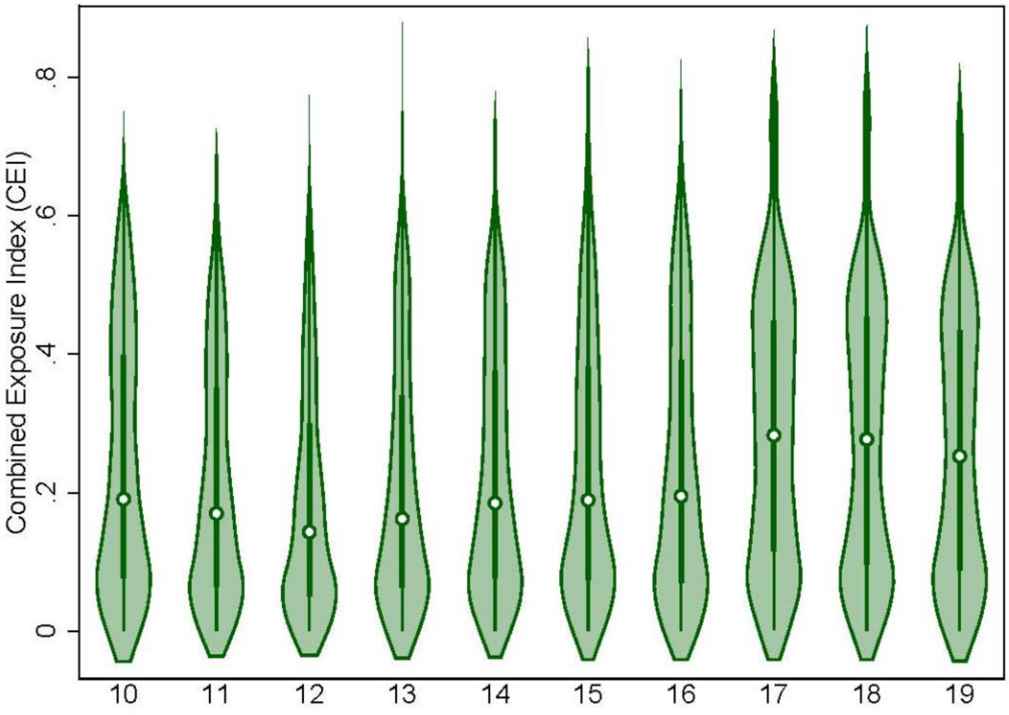

Figure S4. Violin plots showing the distribution of the combined exposure index for all census tracts that contain an ECHO participant for the years 2010-2019.

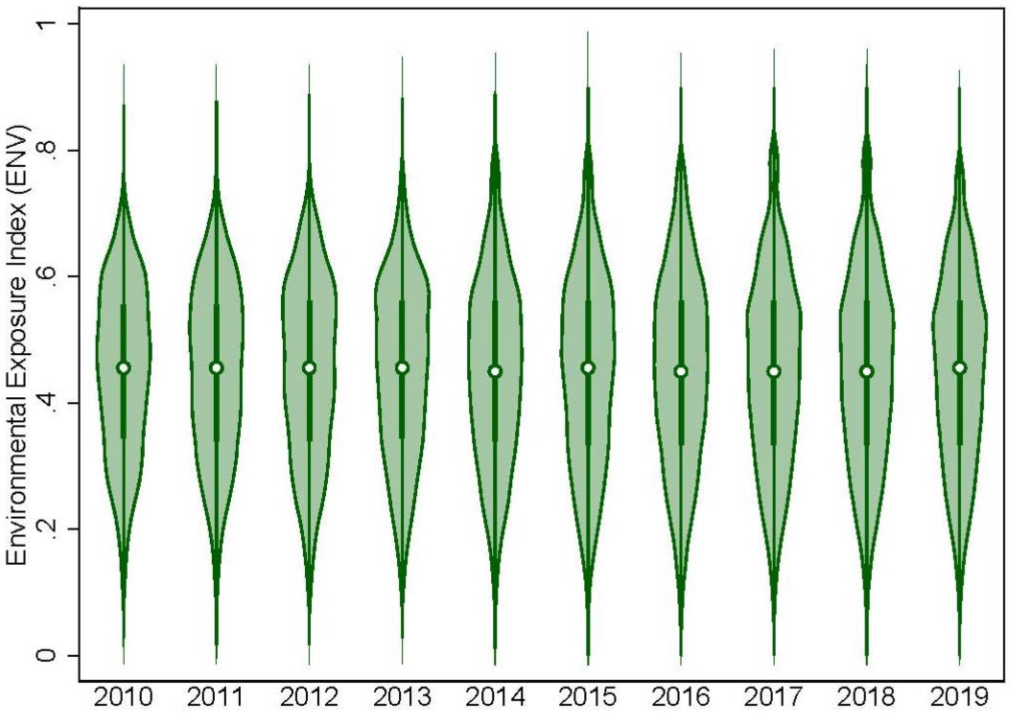

Figure S5. Violin plots showing the distribution of the environmental component score for the United States for the years 2010-2019.

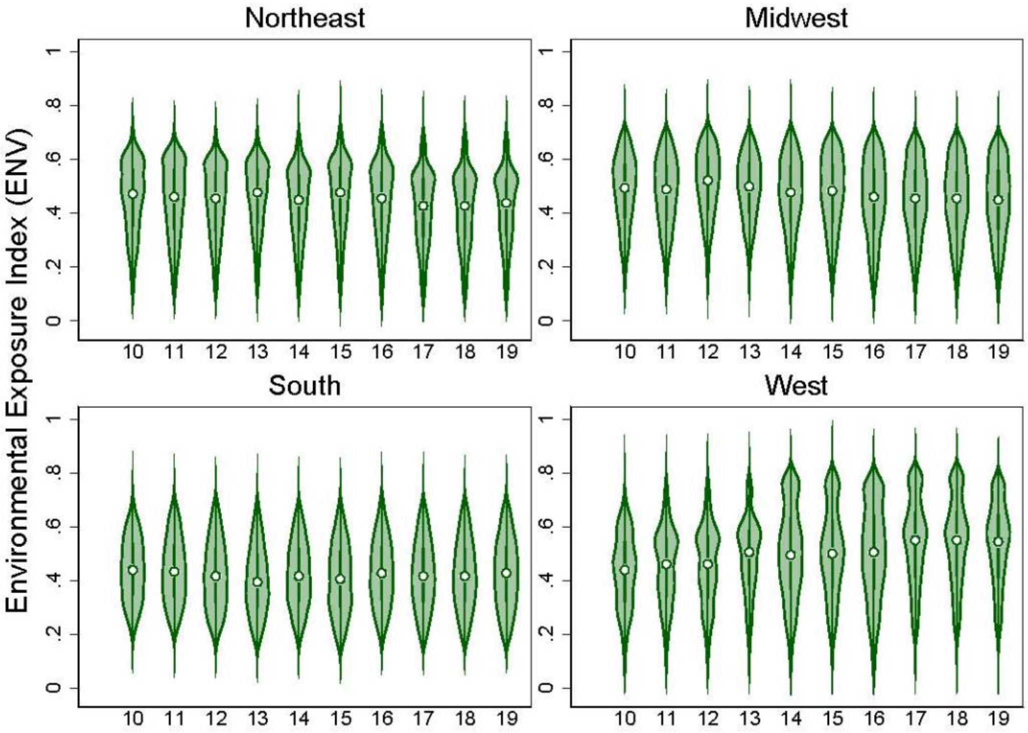

Figure S6. Violin plots showing the distribution of the environmental component score for the United States by region for the years 2010-2019.

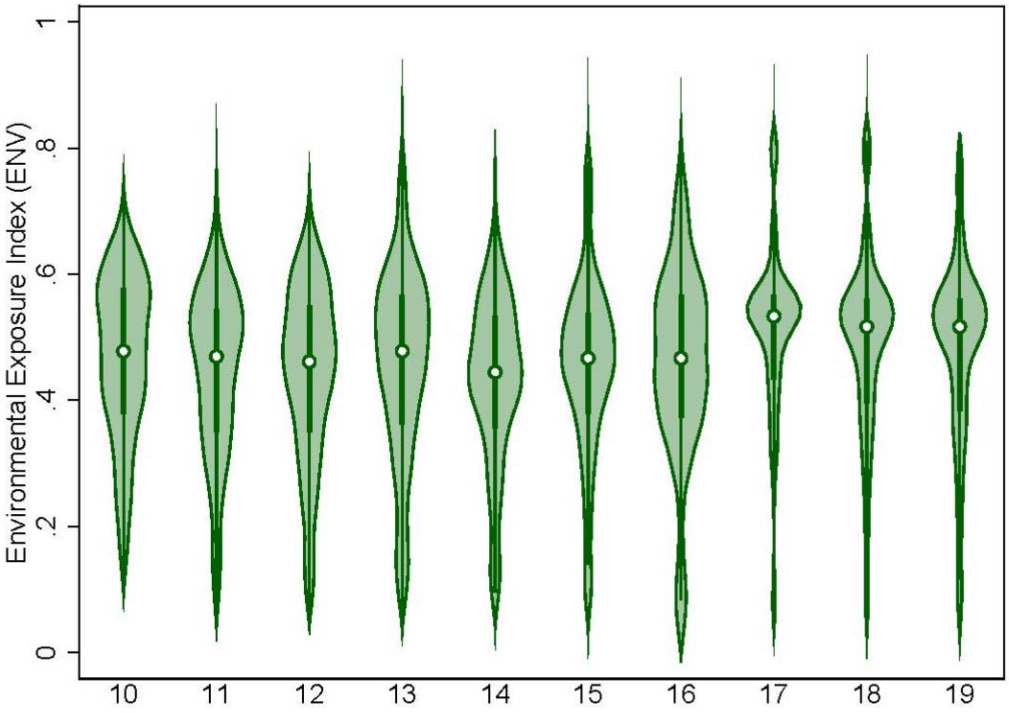

Figure S7. Violin plots showing the distribution of the environmental component score for all census tracts in which ECHO participants lived for the years 2010-2019.

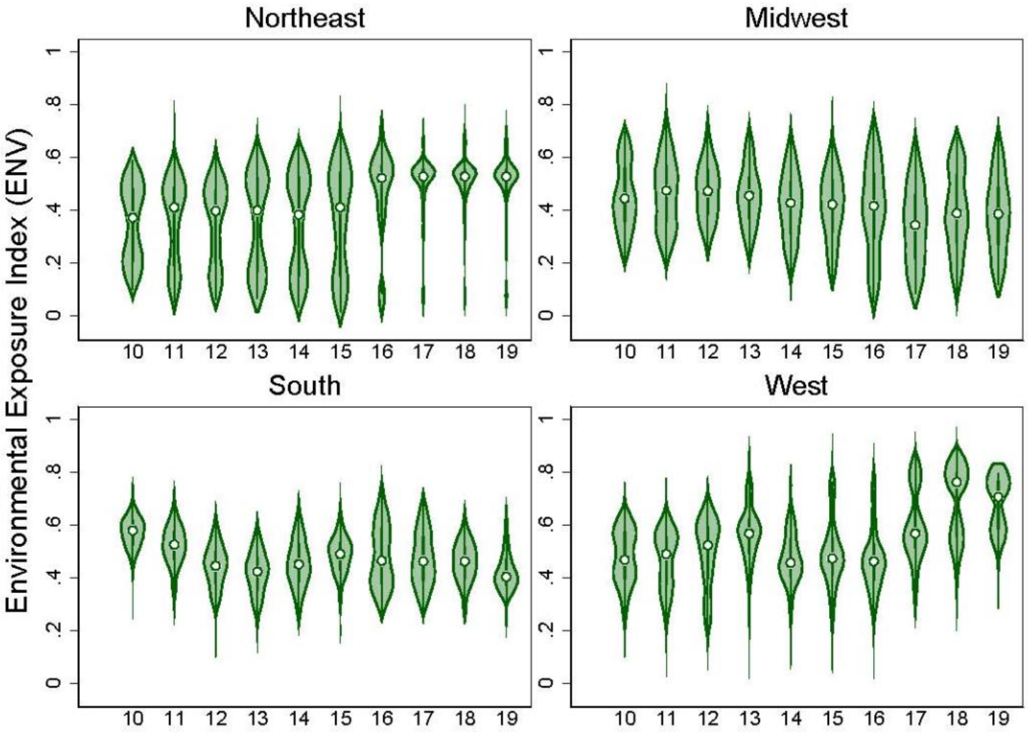

Figure S8. Violin plots showing the distribution of the environmental component score for all census tracts in which ECHO participants lived by region for the years 2010-2019.

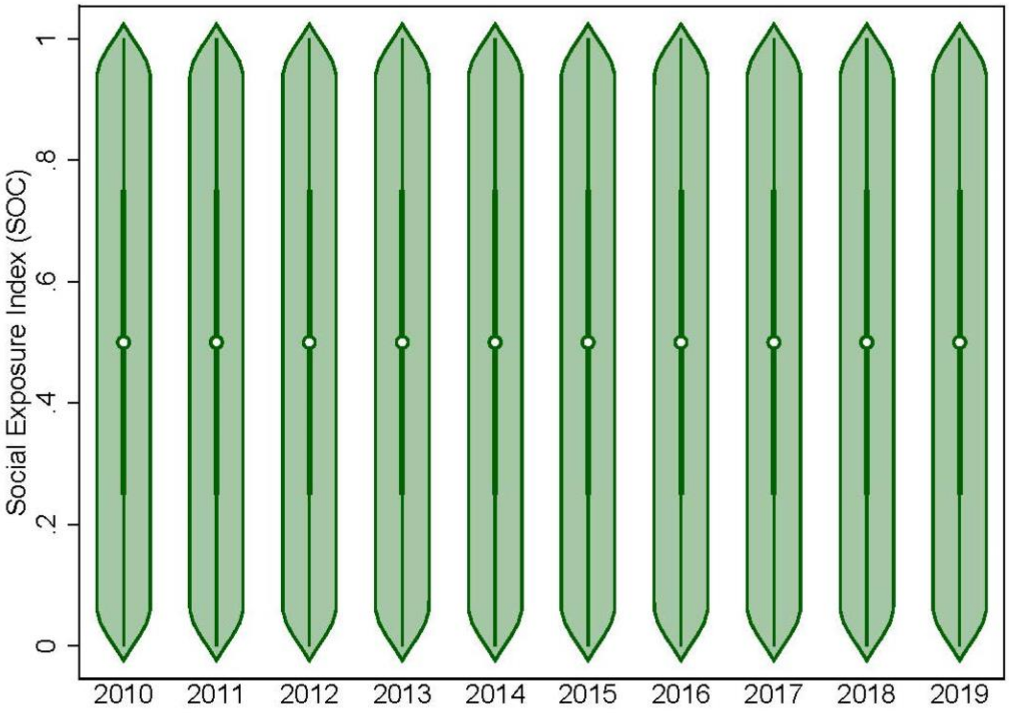

Figure S9. Violin plots showing the distribution of the social component score for the United States for the years 2010-2019.

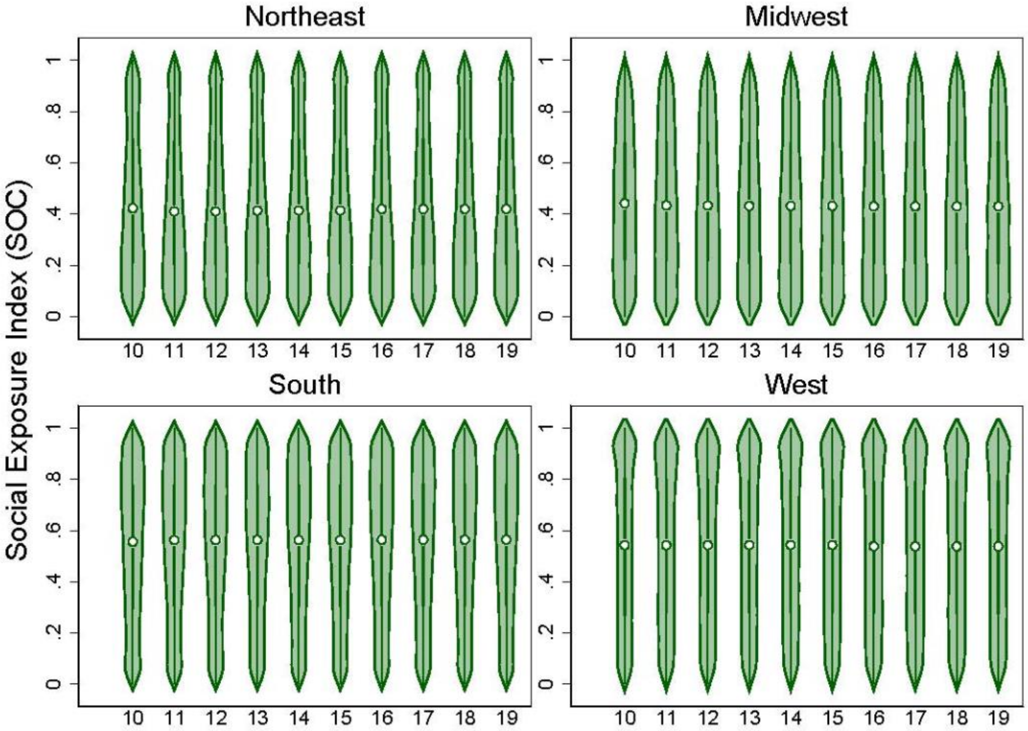

Figure S10. Violin plots showing the distribution of the social component score for the United States by region for the years 2010-2019.

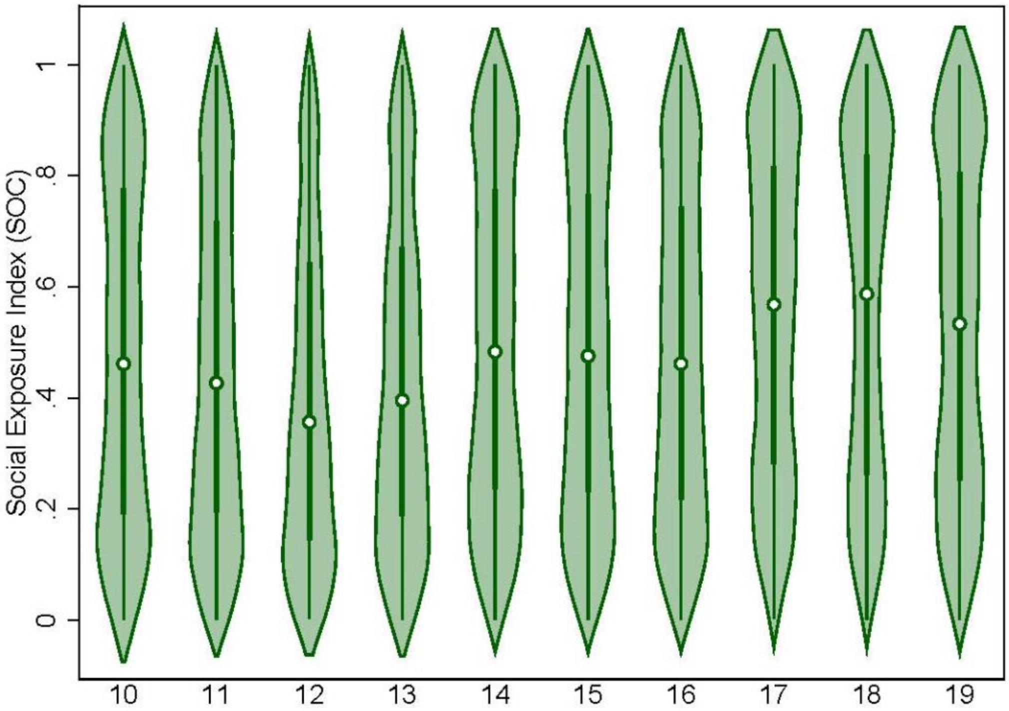

Figure S11. Violin plots showing the distribution of the social component score for all census tracts in which ECHO participants lived for the years 2010-2019.

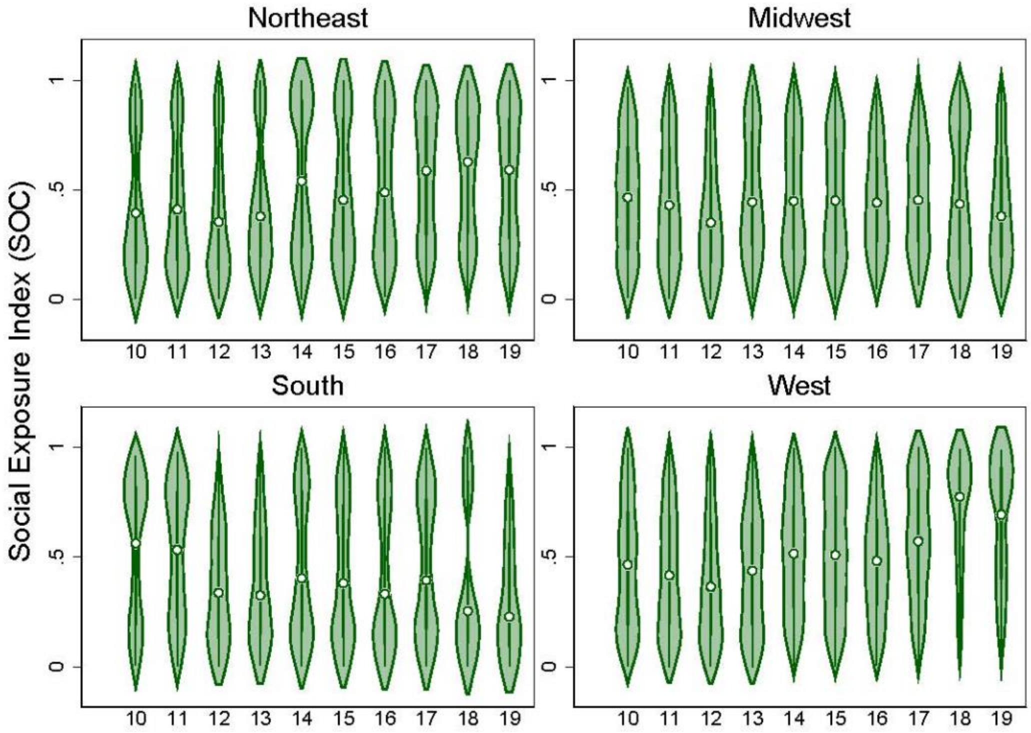

Figure S12. Violin plots showing the distribution of the social component score for all census tracts in which ECHO participants lived by region for the years 2010-2019.

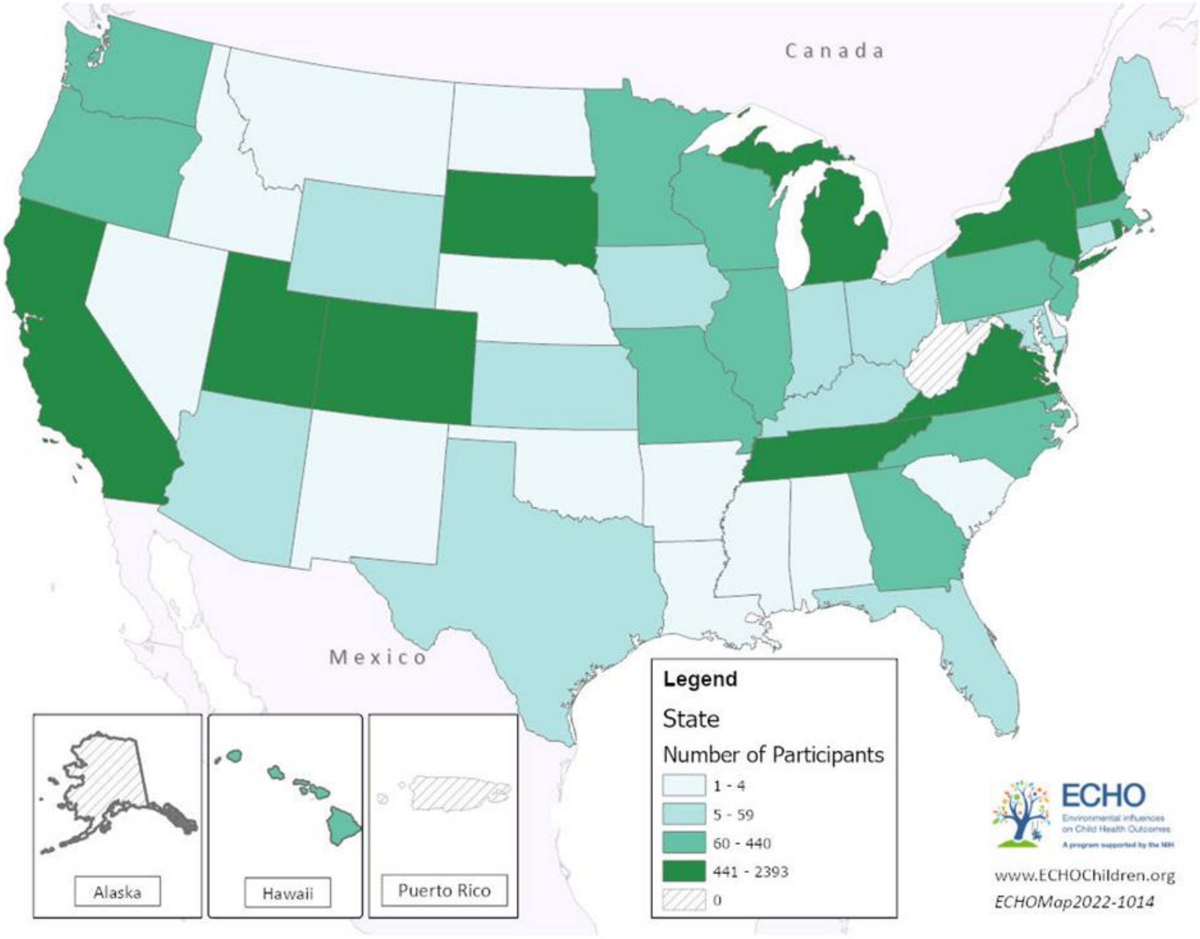

Figure S13. The number of participants by state and region used to assess geographic differences in the combined exposure index.
